# Judging Oneself and the Feedback: Using a Feedback Literacy Lens to Explore How Learners Experience Professionalism Feedback

**DOI:** 10.5334/pme.2320

**Published:** 2026-02-04

**Authors:** Daniela Maristany, Karen E. Hauer, Vincent Grospe, Andrea N. Leep Hunderfund, Martha L. Elks, Bridget C. O’Brien

**Affiliations:** 1 Department of Medicine, University of California, San Francisco, San Francisco, California, US; 2 Department of Medicine, University of California, San Francisco School of Medicine, San Francisco, California, US; 3 University of California, San Francisco School of Medicine, San Francisco, California, US; 4 Office of Applied Scholarship and Education Science, Mayo Clinic College of Medicine and Science, Rochester, Minnesota, US; 5 Morehouse School of Medicine, Atlanta, Georgia, US; 6 Department of Medicine and Center for Advancing Scholarship in Education, University of California, San Francisco School of Medicine, San Francisco, California, US

## Abstract

**Introduction:**

Professionalism is a core competency on which learners should, ideally, receive feedback to improve their performance. Feedback literacy conceptualizes how learners make sense of and use feedback. The contextual and subjective nature of professionalism, along with concerns about professionalism’s potential to encode majority culture norms, add unique complexity to receiving and responding to professionalism feedback. This study used feedback literacy as a framework to explore how diverse learners experience and respond to professionalism feedback.

**Methods:**

The authors conducted a multi-center qualitative study with a critical constructivist orientation. Fourth-year medical students and senior residents were interviewed about their experiences with professionalism feedback. Interviews were analyzed using reflexive thematic analysis. Feedback literacy provided an analytic lens for theme development.

**Results:**

Thirty-one medical students and 18 residents were interviewed between 2021 and 2022. Learners saw little value in professionalism feedback when viewing professionalism as a character trait rather than a skill to be improved. Learners who received constructive professionalism feedback critically reflected on the quality of their own professionalism and of the feedback, specifically evaluating the feedback for racial or other bias. Constructive professionalism feedback generated protracted emotional responses, and learners often lacked agency to respond to professionalism feedback due to the method of feedback delivery.

**Discussion:**

Learners engage with professionalism feedback by spending significant time examining the context of the feedback and searching for evidence of racial or other bias. Understanding how learners experience professionalism feedback is important for fostering strong professionalism feedback literacy for learners and educators.

## Introduction

As a core competency, professionalism requires performance-based feedback to develop and improve learners’ skills [1], but the multifaceted and context-dependent nature of professionalism makes feedback in this domain challenging [2,3,4,5]. While feedback can improve performance [6], the magnitude of improvement varies depending on the mode of feedback delivery and feedback content [6,7]. What makes the professionalism domain particularly complex is the longstanding debate on its definition in medicine [8], with at least three ways of conceptualizing professionalism (e.g., as virtues, as behaviors, as identity) [9]. Correspondingly, agreeing on specific standards that provide a basis for professionalism feedback is difficult.

Prior work on professionalism shows that learners, especially from historically marginalized groups, experience professionalism as enforcing the norms of historically dominant groups in medicine [4,10]. Learners from historically marginalized groups describe feeling they cannot express their identities while still appearing professional and that their professionalism is excessively scrutinized compared to their non-minoritized peers [4,10,11,12]. However, we lack understanding of how the unique sociocultural context of professionalism affects learners’ experiences with feedback or how learners’ responses to professionalism feedback differ from feedback on other competencies. Without this understanding, medical educators may struggle with giving feedback that helps learners improve their professionalism.

Conceptualizations of feedback in health professions education have shifted from viewing feedback as a one-way delivery of information from teacher to trainee [6,13] to a bidirectional conversation [14,15]. Despite this reframing, feedback takes many forms (including formal assessment, informal discussions, unsolicited comments, nonverbal cues [16]) and is not always recognized by learners and teachers as feedback or experienced by learners as helpful [17,18,19]. Learners’ receptivity and response to feedback vary depending on multiple factors. Learners continuously make credibility judgements to determine what information—including feedback—from the clinical environment to integrate or dismiss [20]. Factors that lead to learners accepting feedback include the learner’s perception that the supervisor is clinically knowledgeable, has a good interpersonal relationship with the learner, understands the learner’s role, and is speaking from a position of good will [17,21].

The concept of feedback literacy describes how trainees make sense of feedback and use it to improve [22]. Carless and Boud outline four major features of feedback literacy: 1) appreciating feedback (learners understand the content of the feedback, the purpose of the feedback, and their role in the process), 2) making judgements (learners evaluate their performance in light of the feedback), 3) managing affect (learners handle emotions associated with feedback), and 4) taking action (learners use feedback to inform future performance, which requires both motivation and the opportunity to act on feedback) [23]. Learners’ feedback literacy varies by content, context, prior experience, and personal characteristics, which speaks to the importance of exploring diverse learners’ experiences [23]. Additionally, learners’ interpretations, judgments, emotions, and actions in response to feedback, which comprise feedback literacy, all may be affected by learners’ perceptions of the topic of feedback (professionalism) itself. Feedback literacy provides a lens to understand how learners make meaning of feedback and where barriers to meaning-making and application of feedback arise [24]. As such, feedback literacy may provide insight into challenges and opportunities for feedback on the contextual and socioculturally-nuanced competency of professionalism.

Considering concerns about professionalism definitions, expectations, norms, and feedback, our study explores learners’ experiences of feedback related to professionalism, paying particular attention to experiences of learners from historically marginalized groups. Using feedback literacy as an analytic framework, our research questions ask how learners with diverse identities 1) perceive professionalism feedback and 2) evaluate and respond to professionalism feedback.

## Methods

### Approach

We analyzed a subset of interview data from a larger research project examining professionalism and learner identity broadly [4]. Interview data used in this study, viewed through a critical constructivist orientation [25], explored how learners experienced and interpreted professionalism feedback. Critical constructivism allowed us to focus on how learners make meaning of the feedback inputs they receive, with close attention to how power differentials affect these experiences. Taking this critical orientation was important in answering our research questions given the previously discussed concerns about the ability of professionalism to oppress minoritized learners and encode homogeneity. We used “feedback” to refer to the broad array of experiences that learners absorb in clinical learning environments to understand their performance, including evaluations and assessments, observations of performances of role models, conversations with peers and supervisors, and responses of supervisors and allied health staff to one’s actions [16].

### Setting and participants

We interviewed fourth-year medical students and senior residents in family medicine, surgery, internal medicine, neurosurgery, obstetrics-gynecology, and pediatrics at Mayo Clinic Alix School of Medicine (with 3 campuses in the midwest, south, and southwest), Morehouse School of Medicine, and the University of California, San Francisco (UCSF) School of Medicine. We chose these institutions for their geographic, racial, and cultural/learning climate diversity. The institutional review boards of each institution granted the study exempt status (UCSF 21-35373, Mayo 21-090, Morehouse 1860577).

### Data collection

We developed a semi-structured interview guide as part of a broader project exploring diverse learners’ experiences with professionalism [4]. We asked participants what professionalism in medicine means to them. We queried times they received praise or positive feedback about professionalism and when their professionalism was questioned or they received critical feedback. We probed how praise or criticism of their professionalism affected learners and how they responded. We asked about times when learners saw their identity affecting their experiences with professionalism (see Supplemental Digital Appendix 1). We conducted pilot interviews with 2–3 learners from each site.

We invited learners to interview through standardized emails to listservs for all fourth-year medical students and senior residents. The first author (D.M.) conducted all interviews, lasting 45–60 minutes, over Zoom from November 2021 to November 2022. Participants gave verbal consent before the interview and received a $25 gift card. Interviews were recorded and transcribed using Rev.com, reviewed for accuracy, and de-identified. We used Dedoose to code transcripts and link demographic data to interviews. We interviewed all volunteers to maximize diversity of residency specialty, demographic identities, and intersectional identities.

### Data analysis

In the analysis for our first study from the larger project [4], we had identified all passages where participants discussed feedback. We subsequently analyzed passages addressing feedback using feedback literacy as a sensitizing framework to examine how diverse learners experience and respond to professionalism feedback.

Following Braun and Clark’s approach to reflexive thematic analysis [26], we read all feedback passages to familiarize ourselves with the data. Next, three investigators (D.M., B.O’B., V.G.) inductively coded the data, noting learners’ processing of and responses to professionalism feedback, emotions regarding feedback, and context of feedback discussions. We used Jamboard, a digital interactive whiteboard, to collect and organize codes and met regularly to discuss coding. Consistent with our critical constructivist approach, our analysis focused on how power differentials and sociocultural context affected feedback experiences of diverse learners. Acknowledging the effect of race, gender, and other identities on power and hierarchy in medicine, we noted when identity, or experience as part of a group historically marginalized in medicine, affected the professionalism feedback discussion. We then began developing connections between codes to create initial themes. We made and compared thematic maps to draw connections between themes and returned recursively to the data to review and revise codes and then themes. We organized our themes in relation to the four elements of feedback literacy to help illustrate how learners interpret, evaluate, and respond to feedback on professionalism.

### Reflexivity

As part of reflexive thematic analysis, we examined how our identities and experiences with professionalism feedback affected our analysis and theme generation. We discussed how the identities of the interviewer (D.M.), a cis-gendered Latina/Asian fellow (now clinician educator faculty) may have affected participants’ responses and their comfort or discomfort discussing their own identities and professionalism feedback experiences. Her position as a fellow may have appeared to some participants as a comfortable near-peer, but to others, especially students, her status may have appeared closer to that of an attending or leadership figure and thus inhibited open sharing. Other investigators included a medical student, education researcher, and clinical educators whose racial identities included Filipino and White.

## Results

**Figure 1:**
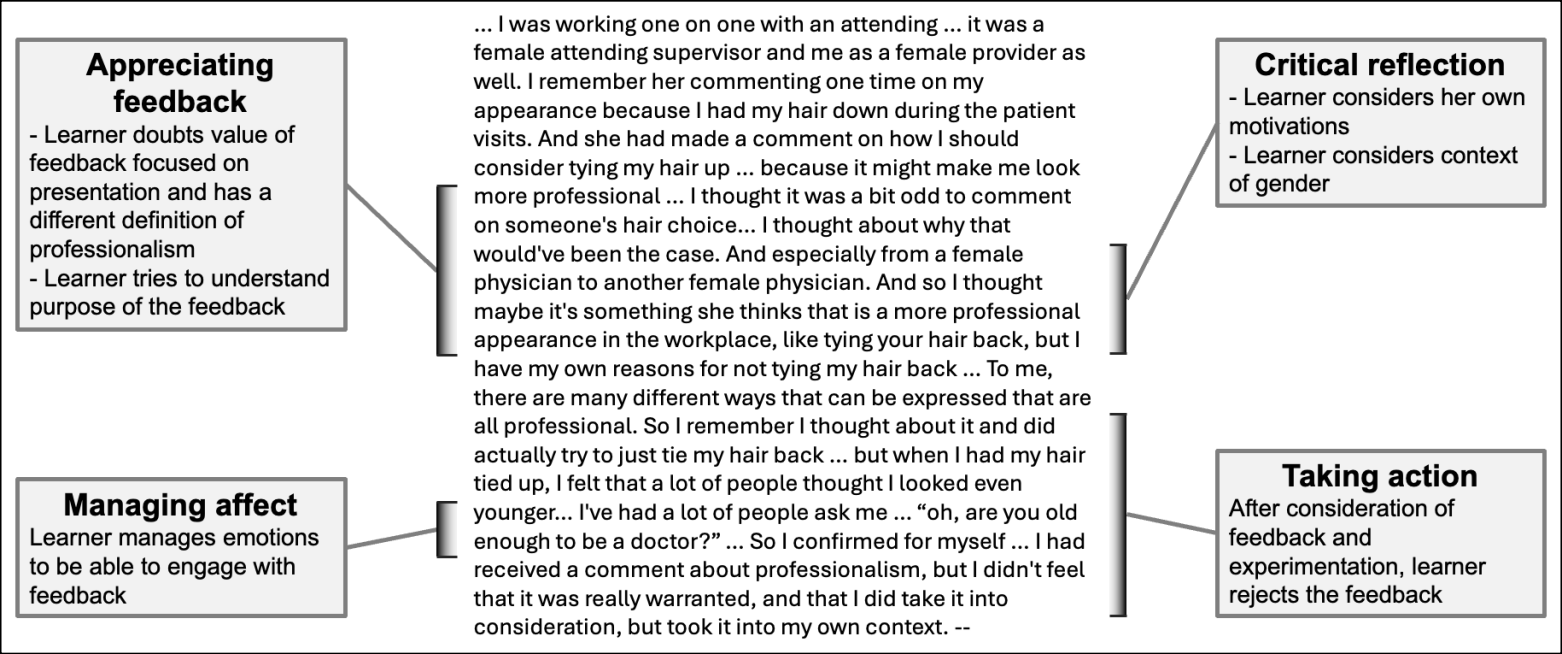
Four features of feedback literacy from a resident describing a professionalism feedback scenario.

We interviewed 49 trainees (31 fourth-year medical students, 18 senior residents) (Table 1); 17 participants identified as under-represented in medicine (URM) and 15 as non-URM persons of color. Using the four key features of feedback literacy (appreciating feedback, making judgements, managing affect, taking action) to frame our results, we present themes within each feature that describe how learners experience professionalism feedback. Figure 1 demonstrates the four features of feedback literacy through the example of a resident describing professionalism feedback. We use the term “historically marginalized,” to refer to groups of people who, due to societal power structures, have not been historically included in medicine as a profession, including women, people of color, people who do not identify as cis-gendered or straight, among others.

**Table 1:** Participant Characteristics (n = 49) of Medical Students and Residents Interviewed about Professionalism at 3 Medical Schools in 2021–2022.^a^


DEMOGRAPHIC	NUMBER (%)

Institution	

Mayo Clinic	24 (49%)

Morehouse	7 (14%)

University of California, San Francisco	18 (37%)

Level of Training	

4^th^ year medical student	31 (63%)

Senior resident	18 (37%)

Specialty (n = 18 residents)	

Family Medicine	3 (17%)

General Surgery	1 (6%)

Internal Medicine	7 (39%)

Neurosurgery	1 (6%)

Obstetrics and Gynecology	2 (11%)

Pediatrics	4 (22%)

Race/Ethnicity	

Asian or Pacific Islander	10 (20%)

Black or African American	10 (20%)

Latinx	3 (6%)

White	16 (33%)

More than one race/ethnicity^b^	9 (18%)

Prefer not to respond	1 (2%)

Representation in medicine	

Underrepresented in medicine (URM)^c^	17 (35%)

People of color, not URM^d^	15 (31%)

Gender	

Cis Women	28 (57%)

Cis Men	21 (43%)

Sexual Orientation	

LGBTQ (lesbian, gay, bisexual, transgender, queer)	8 (16%)

Straight	39 (80%)

Unsure or prefer not to respond	2 (4%)


^a^Reproduced from Maristany D, Hauer KE, Leep Hunderfund AN, et al. The Problem and Power of Professionalism: A Critical Analysis of Medical Students’ and Residents’ Perspectives and Experiences of Professionalism. Acad Med. 2023;98(11S):S32–S41 [4]. ^b^Includes participants who self-identified as multiple races/ethnicities including Asian, Black, Latinx, Middle Eastern, and White. ^c^Includes participants who self-identified as Black/African American, Hispanic/Latinx, Native American/Alaskan Native, Native Hawaiian/Pacific Islander, Filipino, Hmong, or Vietnamese. ^d^Includes participants who self-identified as a race/ethnicity other than White which is not included in the definition of URM (c).

### Appreciating feedback: Learners question the value of professionalism feedback

While learners generally felt professionalism was important, they did not always see the value of professionalism feedback. Learners doubted the value of professionalism feedback because they disagreed with the feedback they received (feeling it reflected a different understanding of professionalism from their own) or believed that professionalism was not a skill requiring feedback. When learners did appreciate the value of professionalism feedback, they were able to identify the clinical or professional purpose of the feedback.

Many learners, especially those from historically marginalized groups, felt the professionalism feedback they received was not appropriate to give in the first place, particularly if the feedback focused on dress, presentation, or identity (see Figure 1). The anticipation of professionalism feedback about their physical presentation created a diminished view of the value of professionalism feedback. For example, a student contrasted what they viewed as professionalism (“I pride myself on my communication and interactions with patients”) with how they felt their institution defined professionalism (“anyone that’s patient facing has to be in full attire of suit, full formal dress wear”). The discrepancy diminished the value of professionalism feedback (“it just feels very rigid and restrictive in some ways and wholly unnecessary”).

Learners’ doubts about the value of professionalism feedback were also fueled by views of professionalism as a character trait or virtue, rather than as a skill they could improve. Several learners noted that they had “thankfully not” received constructive professionalism feedback. Some learners found reinforcing professionalism feedback motivating during challenging periods in training, but many did not find significant value in positive professionalism feedback because it seemed superfluous, inauthentic, or vague. Learners often viewed professional behavior as basic expectations, not praiseworthy. A resident described feeling saddened by positive professionalism feedback for “doing very bare minimum, normal-people things.” Some learners appeared to conflate a lack of negative professionalism feedback with positive feedback, as they replied to the interview question about receiving praise or positive professionalism feedback by stating that they had never been flagged for a professionalism lapse. Learners who did receive reinforcing professionalism feedback often described it as generic (“like a copy and paste thing”) and lacking concrete behavioral examples.

Every so often I get feedback that I’m professional, and I honestly don’t know where it comes from. I feel like it’s more because if the people like you and you don’t have anything obvious that is off, they’ll be more likely to say “Super professional. Communicates well.” … then you’re just wondering what happened if they do say something negative. – Participant 37 (ciswoman, Black, student)

Learners did see the value of professionalism feedback when they could clearly identify the feedback purpose. For example, a resident received feedback from her program director that her email tone to clinic support staff had come across as unprofessional, and the resident noted “it was appropriate feedback because of course you don’t want to alienate your staff.” Another resident reflected that feedback from her attending about not speaking within earshot of a patient’s family was “fine, it was effective, because I needed that reminder” to remain respectful to patients. In contrast, when an attending indirectly questioned whether a resident was inflating his patient admission numbers, the resident dismissed the feedback as “not constructive, it’s more like gossip” and wondered “what is the ultimate goal of [feedback] like that?”

### Making judgements: Learners consider context and bias when judging professionalism feedback quality

In response to constructive feedback, learners judged their own professionalism through prolonged, multidimensional self-reflection. They replayed the event on which they received feedback, considered their own motivations and actions, attempted to understand the feedback giver’s perspective, sought practical or patient-centered implications, and solicited second opinions from mentors. A student described receiving a written evaluation from an attending that described his questions about whether a patient should receive medical or surgical management as unprofessional:

…I thought I was being professional and just trying to understand better why would you choose one [intervention] versus the other, but [the attending] didn’t perceive it that way. He perceived it as a challenge or at least it seemed like a challenge to his authority…I spoke with a few trusted mentors about this. I kept trying to replay it back in my head over and over again. I kept trying to talk to other people like, “Am I like this?”—Participant 44 (cisman, Black/Latino, student)

Learners also made judgements about the quality of the professionalism feedback by considering context (hierarchical, racial, gender, institutional) and potential bias. Learners discounted feedback seeming solely to reinforce the medical hierarchy. One student described:

I was meeting my definition of professionalism. I was doing the most good for the most people… Her [the attending’s] definition of professionalism clearly had a lot more to do with keeping your head down and obeying the person who is a rung above you on the totem pole.—Participant 4 (cisman, Black/Middle Eastern, student)

Learners considered context, including institutional and gender, when examining professionalism feedback and how a professional action at one institution might be considered unprofessional in another. For example, a resident described receiving feedback early in intern year that his interaction with a patient might seem unprofessional and considered how that feedback might reflect the differing cultures of his current institution compared to his medical school:

My perception of the patient encounter was totally fine based on what I had observed for all of those years in medical school where I feel like conversations with patients were a little more frank or patient-physician interactions tended to be a little more casual than where I’m currently training.—Participant 29 (cisman, White, resident)

Gender was another context through which learners interpreted professionalism feedback, especially feedback addressing appearance or demeanor. They considered both their gender and the gender of the feedback giver to interpret why the feedback was given and whether they agreed with the content. Figure 1 describes the thought process of a resident who received feedback from a woman attending about her hairstyle. Another resident recalled observing a female co-resident receive feedback from attendings that her communication style with a patient had been unprofessional: “she thought she was being professional and other people didn’t. I question if it had been a male, if things had gone even slightly differently.”

Learners also considered intersecting identities and nuances in their reflections on whether feedback contained bias. For example, a White-presenting Latino resident considered how race and gender may have influenced the positive professionalism feedback he received and the negative professionalism feedback his co-resident, a woman of color, received for similar actions. A Black student reflected on how feedback on her clothes from her mentor, also a Black woman, was biased against larger bodies but also may have stemmed from holding higher standards due to their shared racial identity. A student considered how negative professionalism feedback from an attending may have been influenced both by the attending not knowing of the student’s extensive prior work experience in the medical field and by the attending’s desire to assert his seniority. When learners perceived bias, or that the professionalism feedback was linked to an element of their identity, they judged the feedback as less valuable.

Learners also evaluated positive feedback for bias. For example, a woman medical student wondered whether positive feedback about her patient rapport and communication was due to her gender. While learners from historically marginalized groups most often considered how race may affect the quality of professionalism feedback received, learners from majority groups also considered the effects of race and identity on the amount of professionalism feedback received:

Probably the fact that I haven’t received explicit feedback about my professionalism is information enough because I make mistakes, we all do. I don’t think I’m more professional than any of my colleagues who might be getting called out for it more than I am. –Participant 47 (ciswoman, White, resident)

### Managing affect: Professionalism feedback feels personal and generates strong, long-lasting emotional responses

Learners often had strong and protracted emotional responses to professionalism feedback because they viewed it as commentary on their character or personality. The idea of professionalism as a baseline expectation further reinforced this characterological frame and exacerbated the subsequent emotional toll.

Those sorts of comments often hit very hard because it feels like it is at the core of who you are. And so it can sort of change the way you see your identity… I think it was about learning how to process that negative feedback, especially around something where it can be hard to talk about and it can feel more personal when you get feedback on your professionalism… it may be harder to respond to it or you may feel at least like, “Oh, it’s a flaw in me more than it is something that I can work on or grow from…”— Participant 43 (cisman, White/Latino, resident)

Views of professionalism as a fixed character trait further contributed to the strong emotions generated by professionalism feedback. When learners saw professionalism as an immutable trait, they interpreted feedback regarding unprofessionalism as putting their entire character into question. A student described feeling upset that he had been labeled as unprofessional due to not turning in a pre-clinical writing assignment:

That was just really infuriating to me because it felt… really bad for somebody to be telling me that they thought I was unprofessional, especially because it feels like a label like, “We’re now seeing this student as unprofessional as opposed to his action was unprofessional.” It feels like more of a character judgment… I actually am really professional.— Participant 1 (cisman, Black, student)

Professionalism feedback also generated protracted responses. Learners described strong affective responses while awaiting the opportunity to discuss the feedback with a supervisor and after the discussions. One student recalled receiving an email about professionalism concerns: “waiting to discuss it really gnawed at me and was a tough 24 hours [until the meeting]… that stuck with me for weeks to, here we are, months later.” After receiving a negative written evaluation about her professionalism on a clinical rotation, a student remembered “the six months of anguish I went through after receiving that negative feedback comment… feeling really demoralized.” A resident recalled receiving feedback as a medical student about his “direct nature,” which was perceived as disrespectful to a supervisor. After this incident, he asked all his subsequent attendings for feedback on his communication style until one attending during residency suggested that he move past the earlier comment.

### Taking action: Learners lack agency to engage with professionalism feedback and may reject feedback that feels biased

Learners often lacked agency to respond to constructive professionalism feedback. Sometimes learners could not act because the professionalism feedback was too vague (“Is my professionalism not mature enough? What does that even mean?”). Other times, learners lacked the platform to engage with the feedback, either because the feedback came through anonymous evaluations or they felt uncomfortable discussing the topic with supervisors. Learners described a desire to talk through feedback with the feedback giver or another supervisor, either to defend their actions or to better understand the comments.

It would’ve been helpful if in that moment they were like, “hey, are you okay or I noticed this, is there something I can do to help?” Just acknowledging it so that I could provide context or they could have better understanding as opposed to just waiting to put that on my evaluation when I can’t do anything about it.” – Participant 25 (ciswoman, Black, resident)

Perceptions that professionalism standards were unattainable either due to bias or unrealistic standards contributed to learners’ feelings of futility in responding to constructive professionalism feedback. A Black student described how the professionalism feedback she received stemmed from a lack of shared backgrounds with the feedback giver, thus she felt “…like there was nothing I could do to achieve the best possible results… if you’re going to do all of this work and not get positive results, then what are you even doing?” A surgical resident described feeling that the residency’s definition of professional behavior (timely response to emails regardless of the clinical rotation the resident is on) did not align with realistic expectations of resident duties:

… you sort of get dinged on the professionalism scale, but it’s not something that you can really fix or do anything about. And I think it creates this sense of futility. And then you think, “Okay, well what’s the point of being professional? Or [what’s the point of] how the institution is defining professionalism if there’s nothing I can do about it? And I’m just going to keep getting blamed for these things that I can’t change.”— Participant 45 (ciswoman, White, resident)

After engaging in critical reflection to evaluate one’s own actions and the feedback quality, sometimes learners determined that the most appropriate action was to reject professionalism feedback (see Figure 1). Learners were more likely to discard feedback that appeared unrealistic (e.g. residents in day-long surgical cases told to answer email more promptly), racially biased, or not applicable to patient care. A Black student received feedback from an attending that he was speaking too informally with patients with whom he shared a racial identity and ultimately decided to reject that feedback:

… these were patients with whom I had shared identities and so I think that sort of natural rapport and familiarity sort of came out…I think that that’s something that maybe these attendings who didn’t share in those identities couldn’t really understand… It just was outside their idea of what being a professional is, and so I think I was just kind of like, “Okay. Thank you for the feedback, but I know that whatever I did in that room worked with that patient and I would do it again, so I’m going to leave that one.”— Participant 1 (cisman, Black, student)

The decision to dismiss feedback often occurred only in the learner’s mind, without verbal discussion between the learner and feedback giver.

## Discussion

Our study describes how diverse medical students and residents interpret and respond to professionalism feedback, using the lens of feedback literacy. We demonstrate how the four elements of feedback literacy (appreciating feedback, making judgements, managing affect, and taking action) manifest uniquely when learners navigate professionalism feedback. Our study builds upon our prior work about professionalism’s power to encode majority culture norms [4] and provides new learner perspectives on professionalism feedback while considering context and identity. Learners often did not value professionalism feedback because they felt it focused on aspects such as dress or presentation that were not pertinent to professionalism. In the few examples where learners valued professionalism feedback, they understood and appreciated the purpose of the feedback. They assessed whether the professionalism feedback was biased (based on race, gender, or other identities) and were likely to reject feedback that appeared biased. Learners were often unable to act in response to professionalism feedback because the feedback was given in a dyssynchronous manner or because the idea of professionalism as a character trait made improving one’s professionalism seem daunting. Because professionalism was closely linked to character in learners’ minds, feedback on professionalism felt highly personal and generated lasting emotions.

**Figure 2:**
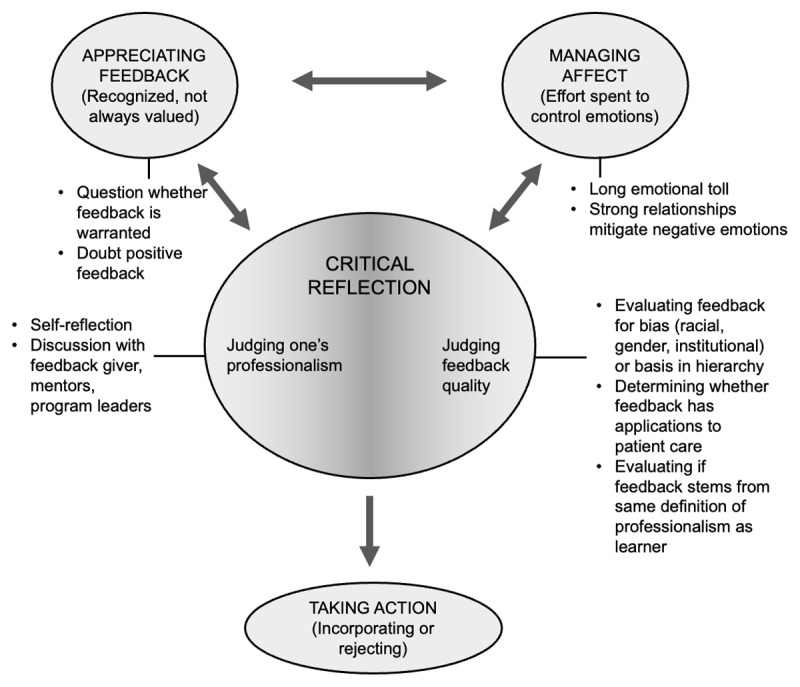
Proposed model for learner responses to professionalism feedback using Carless and Boud’s framework for feedback literacy^23^ (see main references list: 23. Carless & Boud).

Our findings show how, for feedback on professionalism, learners spend a disproportionate amount of time making judgements. Carless and Boud describe this element of feedback literacy as evaluating the quality of one’s performance [23], but we found that for professionalism feedback, learners also spend significant effort judging the quality of the feedback and determining whether to integrate or ignore it. This process generally occurred simultaneously with making judgements about their own professionalism and managing the emotional response to feedback (Figure 2). Watling [20] and Telio [27] have demonstrated how learners make credibility judgements to determine which feedback from the learning environment they take or discard. We found a unique type of learner credibility judgement—searching for evidence of racial or other bias in both constructive and reinforcing professionalism feedback. Outsized emphasis on evaluating professionalism feedback for bias may consume excessive time and energy, thus contributing to learner burnout. However, this process can also teach critical reflection and decrease hierarchy if learners are encouraged to evaluate and question the professionalism feedback they receive. Truly empowering learners to assess and critically reflect on professionalism feedback will require humility and openness on the part of educators to acknowledge that the feedback they give is not always specific or actionable and may be biased. Professionalism curricula can be enriched by acknowledging the value of critical reflection and teaching frameworks for reflection to encourage learners to evaluate feedback quality as part of their response to feedback.

The common view among learners, as well as educators [9], that professionalism is a character trait rather than a skill to grow, poses challenges to successful engagement with professionalism feedback. Our findings show how this character-based definition of professionalism affects all elements of learner professionalism feedback literacy, from learners not appreciating the need for professionalism feedback to a feeling of futility when faced with the need to improve their professionalism. Addressing this mindset will require work by institutions, professionalism curricula, and individual faculty, including faculty modeling a growth-mindset for professionalism. Institutions can support a shift away from character-based professionalism by presenting professionalism as a skill to continuously work on rather than a code of conduct to be adhered to.

While all feedback can feel personal and emotionally charged [28,29,30], feedback about professionalism is especially prone to producing strong emotions given the association of professionalism with traits and virtues [9]. While emotions have often been viewed as an inhibitor to feedback and feedback literacy [31,32], Ajjawi [33] and Molloy [28] argue that emotions are inextricable from feedback and should be considered and learned from rather than suppressed. Given our study’s demonstration of the consistent emotional response provoked by professionalism feedback, strong emotions should be viewed as a valid response to professionalism feedback, especially feedback that may feel biased. Institutions can enrich professionalism curricula by including discussions of the anticipated emotional response to professionalism feedback and how to engage with, rather than subdue, emotions.

Our data demonstrate the barriers facing learners in responding to professionalism feedback but also present opportunities for improvement. The dyssynchronous nature of most professionalism feedback and the prolonged process of evaluation that learners engage in after receiving constructive professionalism feedback present challenges to discussing the feedback in the moment. Medical schools and residencies can address the lack of opportunities to engage with professionalism feedback by providing follow-up verbal discussions of written professionalism feedback and structured opportunities for learners to debrief about the professionalism feedback they receive. For these debriefs to be effective, programs will need to provide scaffolding to manage the power differential and thus allow for dialogue between learner and teacher. Such scaffolding may involve having the discussions occur with a trusted coach and faculty development on creating open learning environments for challenging conversations.

Our findings also have implications for faculty development and teacher feedback literacy, an important accompaniment to learner feedback literacy [34]. Teachers may eschew giving direct professionalism feedback for multiple reasons, including discomfort, feeling unprepared, and wanting to save face for learners [35,36]. Our findings suggest applications for the relational dimension of teacher feedback literacy [34], as teachers who appreciate and acknowledge the potential for bias in professionalism feedback may improve feedback experiences and prevent harmful feedback. Faculty development on feedback practices needs to include discussion of the unique challenges of giving professionalism feedback so that faculty understand that learners are evaluating feedback for evidence of bias and are more appreciative of the value of professionalism feedback when the purpose of the feedback is clear. Additionally, given the potential for bias in professionalism feedback, medical educators need training and resources to evaluate the feedback they plan to give for bias. Educators must accept that a viable learner response to professionalism feedback may be to reject it. Such training will also help educators reframe their assessment of learners’ feedback literacy. Rather than demonstrating a lack of feedback literacy, learners’ actions may reflect legitimate concerns about the value or quality of professionalism feedback, difficulty interpreting and responding to the feedback, or contextual or environmental challenges to feedback [37,38,39].

Our study has limitations. The majority of study participants were from UCSF and Mayo, were medical students, and identified as straight; all participants identified as cis-gendered. Thus, our findings do not speak to the unique professionalism experiences of learners who come from other institutional contexts or identify differently. One researcher conducted all interviews, which improved uniformity but also meant that the interviews may have been influenced by that researcher’s unconscious biases and views on professionalism. Our findings may also reflect participation bias, as volunteers for interviews may have had especially strong views on professionalism. Learners with distressing professionalism feedback experiences during training may have declined participation to avoid sharing traumatic memories. Additionally, as the study data came from semi-structured interviews about professionalism broadly, a consistent description of when and where the feedback was delivered was not available for all the anecdotes about professionalism feedback; such details could illuminate further why learners responded to feedback in the described ways.

## Conclusion

Our study demonstrates that learners respond to professionalism feedback by critically reflecting on the quality of their own professionalism and of the feedback received. Learners judge professionalism feedback quality by considering context and searching for bias. Learners’ responses to professionalism feedback include strong emotions provoked by the view of professionalism as a character trait rather than a skill. To address the sociocultural contexts of professionalism and feedback literacy, medical educators need to support learners to evaluate professionalism feedback and support faculty to deliver actionable, behaviorally-based feedback that minimizes bias.

## Previous presentations

Versions of these results were presented at the Academy for Professionalism in Health Care online conference (November 2023) and the University of Southern California Innovations in Medical Education online conference (February 2024).

## Additional File

The additional file for this article can be found as follows:

10.5334/pme.2320.s1 Supplemental Digital Appendix 1. Interview Guide Used to Conduct Semi-structured Interviews on Professionalism with Students and Residents at 3 Medical Schools in 2021–2022.pdf.
