# Supplementary material for: Judging Oneself and the Feedback: Using a Feedback Literacy Lens to Explore How Learners Experience Professionalism Feedback
Source: Perspect Med Educ. 2026 Feb 4;15(1):93–104. doi: 10.5334/pme.2320 (PMC12880047; doi:10.5334/pme.2320)
Supplement: Supplemental Digital Appendix 1. — Interview Guide Used to Conduct Semi-structured Interviews on Professionalism with Students and Residents at 3 Medical Schools in 2021–2022.pdf. [file pme-15-1-2320-s1.pdf]

Supplemental Digital Appendix 1
Interview Guide Used to Conduct Semi-structured Interviews on Professionalism with Students and Residents at 3 Medical Schools in 2021-2022

Interview Guide

Thank you for meeting today to share your perspective on professionalism in medical education. I am [name] and [brief description of position and role in this project]. In this interview, I will ask you to tell me your views on medical professionalism – what it means to you, what you perceive as its purpose and value, your impression of how it's taught and assessed in your medical school or residency program, and how you've personally experienced professionalism. We are interested in stories or specific examples of your experiences with professionalism.

I shared an information sheet with you and want to make sure you have had time to review it. Would you like time to review? Do you have any questions about the information or about participating in this study?

Is it okay with you if I begin recording this interview? I would like to remind you that you are free to stop the interview at any point or to ask me to stop the recording. (if Yes, begin recording)

Today is (DATE) and this is an interview with (Participant ID).

Can you confirm what year in medical school or residency you are in?

Questions

1. What does professionalism in medicine mean to you?
    - [Probe] How is professionalism in medicine different from professionalism in general?
2. Can you tell me about a time when you observed or participated in something you considered the essence of professionalism or a prime example of professionalism?
    - [Probe for details if not given] What was the setting, who was involved (no names), what happened, how was the professionalism recognized (if at all), how did this affect your understanding of professionalism?
    - [If initial answer doesn't elicit this] Can you recall a time when you observed or participated in something you considered consistent with professionalism, but others did not consider it consistent with professionalism? Who was involved (no names), what happened, how did this affect your understanding of professionalism?

3. Now can you tell me about a time when you observed or participated in something you considered questionable in terms of professionalism or something that you considered potentially unprofessional?

    - [Probe for details if not given] What was the setting, who was involved (no names), what happened, how did this affect your understanding of professionalism?
    - [If initial answer doesn't elicit this] Can you recall a time when you observed or participated in something you considered questionable professionalism, but others did not find it questionable?

With these examples in mind, I'd like to discuss your understanding and experiences of professionalism more generally.

4. Can you tell me about a time when professionalism has been useful in your medical training and/or your clinical work? Please explain.
    - **Have you received praise or positive comments on your professionalism during medical school or during residency?**
        i. **Tell me about that experience (what was the setting, who praised you, what did they praise you for, how did it happen, did the praise resonate with you/did you feel it was appropriate).**

5. Can you tell me about a time when professionalism interfered with your medical training and/or your clinical work?
    - **Can you think of a time when your professionalism was called into question, or you received critical feedback on your professionalism during medical school or during residency?**
        i. **Tell me about that experience (who doubted/criticized you, what did they criticize you for, how did it happen, did the criticism resonate with you/did you feel it was appropriate).**

6. What do you think is the patient's responsibility to the professional interaction or to professionalism in medicine?
    - Can you think of a time when you feel like patients have not upheld their responsibility to physicians / medicine?

I'd like to turn now to ask more about your experience of professionalism from the perspective of your identities, including race/ethnicity, gender, and any other identities you hold.

General questions:

7. How do you think professionalism impacts you in particular?
    - How do you feel your experience of professionalism is affected by your identity?

Specific

8. **Can you share an example of a time that someone's views about your professionalism seemed influenced by your race/ethnicity or by a characteristic or aspect of your identity?**
   Or
   Can you share an example where you witnessed views about someone's professionalism were influenced by their race/ethnicity/non-majority group status?

9. Professionalism can also be considered at the level of systems or institutions. How do you see the medical systems you've worked in supporting professionalism? What about inhibiting professionalism? (Make sure to cover both supporting and inhibiting)
   - Are there things that medical systems (health systems or medical schools/residency programs) could or should do to better support professionalism? Could you give me some examples OR tell me more about that?
   - [Not necessary to ask] Are there ways you think professionalism can positively contribute to the functioning of a health system?
   - [Not necessary to ask] Are there ways you think professionalism can positively affect patients or positively affect patient-physician interactions?

10. What do you think has contributed to your understanding of professionalism?
    - Some people think about professionalism as a matter of character or personal values. How do you think character and personal values relate to professionalism?
    - [Not necessary to ask, but listen for comments about rules/standards and follow up] Some people think about professionalism as a set of external rules or standards. What are your thoughts on this view of professionalism? Do you feel a set of external rules has affected how you enact professionalism? If so, how?
    - [Probe if not mentioned in response]: Can you identify any ways that your med school / residency program formally teaches professionalism? Please explain
    - [Probe if not mentioned in response]: Can you identify any ways that your med school / residency program assesses professionalism? Please explain

11. What do you think is the purpose of teaching about and assessing professionalism in medical training?
    - What do you think *should be* the purpose of teaching about and assessing professionalism?

12. Is there anything you’d like to add about professionalism that we haven’t discussed?

Thank you for your participation. I will send you an electric gift card for you participation. [Confirm Email after turning off recording]
